# Social Support Associated with Quality of Life in Home Care Patients with Intractable Neurological Disease in Japan

**DOI:** 10.1155/2012/402032

**Published:** 2012-10-01

**Authors:** Tomoko Nishida, Eriko Ando, Hisataka Sakakibara

**Affiliations:** ^1^Department of Nursing, Sugiyama Jogakuen University, 17-3 Hoshigaoka-Motomachi, Chikusa-Ku, Nagoya 464-8662, Japan; ^2^Division of Health, Bureau of Health and Welfare, 1-1 Sannomaru 3-Chome, Naka-Ku, Nagoya 460-8508, Japan; ^3^Department of Nursing, Nagoya University Graduate School of Medicine, 1-1-20 Daiko-Minami, Higashi-Ku, Nagoya 461-8673, Japan

## Abstract

The aim of the present study was to investigate what kinds of social supports contribute to the higher quality of life (QOL) of home care patients with intractable neurological disease. We investigated the World Health Organization Quality of Life-BREF (WHOQOL-BREF) and social supports to 74 patients with intractable neurological disease in a city of the Aichi prefecture, Japan. Association between WHOQOL and social supports was examined using multiple logistic regression analyses adjusting activities of daily living (ADL). High WHOQOL scores were associated with “attending patient gatherings held by the public health center,” “having someone who will listen empathically to anxieties or troubles,” and ADL. Physical health was associated with ADL, while psychological well-being was related to “having a hobby,” “having someone who will listen,” and “having a hospital for admission in emergencies.” Patients not having someone who will listen were more likely to participate in the gatherings. The present findings suggest that having someone who will provide emotional support is important for home care patients with neurological diseases. Patient gatherings held by the public health center were expected to provide patients with emotional support.

## 1. Introduction

Some chronic progressive degenerative neurological diseases without a cure are called “intractable neurological diseases” in Japan, where patients are provided with welfare services such as support for patient's care and subsidization. The diseases include Parkinson's disease, spinocerebellar degeneration, multiple sclerosis, and amyotrophic lateral sclerosis (ALS). They develop degenerative changes and progressively lead to motor paralysis, sensory impairment, involuntary movement, and muscle weakness/atrophy and increasing levels of physical disability [1]. Thereby the patients suffer not only medical and economical problems but also psychological difficulties. It is shown that the diseases have a significant impact on psychological well-being and quality of life (QOL) of patients as well as their physical well-being [2–6].

Many studies have investigated the factors affecting the QOL of patients with intractable neurological disease [7–10]. Social support was one of the effective factors for QOL and well-being of the patients. A strong positive correlation was found between social support and QOL among patients with MS [11]. Social support had an association with lower depression in patients with Parkinson's disease [12]. On the other hand, some studies reported that negative or unsatisfied social support could produce higher psychological distress [13, 14]. It is, hence, important to examine which social supports are helpful for patients with intractable neurological disease.

Social support is composed of material support, informational support, emotional support, and so forth. Especially emotional support is very important. Sympathy, comfort, and affection from a familiar person may be as necessary as useful information and actual help. The patients receive various supports to live at home from their family, friends, medical professionals, and volunteers. In Japan, public health departments also provide patients with support services, such as information about medical care and welfare, giving advice about the troubles of patients and their family and making an occasion to have contacts with patients. The aim of the present study was to investigate what kinds of social supports contribute to the QOL of home care patients with intractable neurological disease. We investigated the concrete social support by familiar persons, professionals, and public institutions to find some supportive measures to improve the QOL of home-care patients.

## 2. Methods

### 2.1. Subjects and Survey Methods

The present subjects were recruited from home care patients with neuromuscular diseases living in an area of A city in Japan who were entitled to receive public welfare services for the specified thirteen intractable neurodegenerative and neuromuscular diseases in 2005: Parkinson's disease, spinocerebellar degeneration, ALS, multiple sclerosis, multiple system atrophy, myasthenia gravis, Huntington's disease, adrenoleukodystrophy, subacute sclerosing panencephalitis, neurofibromatosis type 1 and 2, prion disease, amyloidosis, and moyamoya disease. The patients who had been in the hospital, had mental deficiency, and were underage were excluded from the study. This left 120 patients eligible of the subjects as in the present study.

Requests for participation in this survey were first sent by post to these 120 people together with certificates of informed consent. Eighty-nine people (74%) returned the certificates of consent for participation, and then an anonymous self-completed questionnaire form was delivered to them and collected by post. There were surveys that were not answered completely. We excluded these from the analysis. Seventy-four people (62%) completed questionnaires, which were used for analysis in this survey. The survey period was from November 2005 until June 2006. This study was approved by the Ethics Committee of Nagoya University School of Medicine.

### 2.2. Survey Contents

The Japanese version of the WHOQOL-BREF was used for QOL measurements of the home patients with intractable neurological diseases. The WHOQOL-BREF is widely used in many countries to assess the QOL of both healthy people and those with some disease, and its reliability and validity have been demonstrated [15]. The Japanese version of the WHOQOL-BREF is a 26-item, self-administered questionnaire developed by Tazaki and Nakane [16]. The WHOQOL-BREF consists of two general questions (on assessment of living quality and satisfaction with health) and four domains: physical health (7 items), psychological well-being (6 items), social relationships (3 items), and environment (8 items). Responses to all the items are made on a scale of 1 to 5. In the tallying process, three items of the 26 questions are negative questions, and so the response scale is reversed for tallying. Then, the total scores of WHOQOL-BREF and each domain are transformed into a 4-to-20. The higher scores indicate a higher perceived QOL.

Six items were evaluated for activities of daily living (ADL): walking, eating, toileting, changing clothes, bathing, and going out. Each item was evaluated on a 4-point scale: “Can do myself,” “Can do with a little difficulty,” “Need partial assistance,” and “Need complete assistance.” According to the responses, the subjects were classified into three groups. People who responded “Can do myself” or “Can do with a little difficulty” to all 6 items were classified in an independent group. People who did not respond “Need complete assistance” to any question but responded “Need partial assistance” to at least 1 item were classified in a low-level care group, and those who responded “Need complete assistance” to at least 1 item were classified in a high-level care group.

In addition to the above, the survey included questions on the disease name under treatment, medical consultation status, frequency of talking with others, frequency of going out, having/not having a hobby, and social supports. Social supports consisted of four dimensions: personal support (having/not having a caregiver, having/not having a person who will listen empathically to anxieties or troubles, and having/not having someone who helps you whenever you are in trouble), community support (join/does not join self-help patient groups and attend/does not attend patient gatherings held by the public health center), medical support (uses/does not use home-visit nursing or rehabilitation service, has/does not have a hospital which will admit you when your condition suddenly changes, and has received/has not received consultations with medical professionals, an easy-to-understand explanation about their diseases, and up-to-date information during medical visits).

### 2.3. Statistical Analysis

The subjects were divided into a high-score group and low-score group based on the median value of the total WHOQOL-BREF score and four domain scores. Survey items associations with the QOL were examined using multiple logistic regression analyses in which QOL score (high versus low) was the dependent variable. First, each survey item was included in the logistic regression analyses adjusting for sex, age, and ADL. Second, survey items that tended to be related (*P* < 0.10) were included in the logistic regression analyses, adjusting for sex, age, and ADL. Same analyses were conducted with each of the total WHOQOL and the 4 domains (physical health, psychological well-being, social relationships, and environment). A chi-square test was used to analyze the relation between survey items and the factors related to QOL in multiple logistic regression analysis. *P* values of < 0.05 were considered statistically significant. These statistical analyses were completed with the statistical software package SPSS 14.0J for Windows.

## 3. Results

The characteristics of the 74 people in this survey are shown in Table 1. There were 38 males (51.4%) and 36 females (48.6%), with ages of 22–80 years and a mean age of 63.9 ± 12.0 (mean ± standard deviation, SD) years. The number of years under medical treatment was 0–28, with a mean of 8.0 ± 5.5 years. Patients with Parkinson's disease accounted for more than half of the subjects (42 patients, 56.8%), followed by 10 patients (13.5%) with spinocerebellar degeneration, and 7 patients (9.5%) with multiple sclerosis. In terms of ADL, 37 of the patients (50.0%) were in the independent group, 15 (20.3%) were in the low-level care group, and 22 (29.7%) were in the high-level care group.

The Cronbach reliability coefficient for all questions on the WHOQOL-BREF was 0.93. The coefficients for each of its domains were physical health (domain 1) 0.83, psychological well-being (domain 2) 0.89, social relationships (domain 3) 0.74, and environment (domain 4) 0.80. These confirm the good internal consistency of the instrument. The mean of the total WHOQOL-BREF score was 11.6 ± 2.4; the physical health (domain 1) score was 11.2 ± 3.2; psychological well-being (domain 2) score was 11.5 ± 3.3; social relationships (domain 3) score was 12.0 ± 3.2; environment (domain 4) score was 12.0 ± 2.3.

Based on the median value of 11.6 in the total WHOQOL-BREF score, the subjects were divided into a high-score group (*n* = 35) and a low-score group (*n* = 39). Logistic regression analyses adjusting for age, sex, and ADL (Table 2) showed significant differences between the two groups in ADL (odds ratio (OR) 2.19, 95% confidence interval (CI) 1.19–4.01), *having a hobby* (OR 3.17, 95% CI 1.30–10.64), *having a person who will listen empathically to anxieties or troubles* (OR 3.29, 95% CI 1.02–10.62), and *attending patient gatherings held by the public health center* (OR 3.83, 95% CI 1.19–12.34). The high QOL score was associated with frequency of going out (OR 1.98, 95% CI 0.97–4.05), though not statistically significant. To clarify factors closely related to QOL score, a multiple logistic regression analysis was conducted with regards to all these factors. As shown in Table 3, significant associations were encountered between the high QOL score and *attending gatherings held by the public health center* (OR 6.07, 95% CI 1.37–26.88) and *having someone who will listen empathically to anxieties or troubles *(OR 6.38, 95% CI 1.31–30.99), in addition to ADL (OR 2.17, 95% CI 1.06–4.43).

Similarly, multiple logistic regression analyses were conducted after adjusting for age, sex, and ADL for each of the 4 domains (Table 4). High physical health (domain 1) was associated with ADL (OR 2.57, 95% CI 1.27–5.20) and *having a person who will listen empathically to anxieties or troubles* (OR 3.42, 95% CI 0.98–11.92). High psychological well-being (domain 2) was associated with *having a hobby* (OR 5.78, 95% CI 1.31–25.52), *having a person who will listen empathically to anxieties or troubles* (OR 8.79, 95% CI 1.17–65.93), *having a hospital for admission* (OR 7.66, 95% CI 1.16–50.36), and *attending patient gatherings held by the public health center *(OR 5.00, 95% CI 0.95–26.32). High social relationships (domain 3) were associated with *not using home-visit nursing or rehabilitation service *(OR 0.04, 95% CI 0.01–0.30) and *having a person who will listen empathically to anxieties or troubles* (OR 4.17, 95% CI 1.04–16.64). High environment (domain 4) was associated with *having a hospital for admission* (OR 5.57, 95% CI 1.29–24.00) and *having a hobby* (OR 3.64, 95% CI 1.10–12.08).

The present study showed an association between the total QOL score and the items of “*having someone who will listen empathically to anxieties and troubles*” and “*attending patient gatherings held by the public health center.*” The characteristics of the factor were further investigated in Table 5. People having someone who will listen empathically to anxieties and troubles were also more likely to have someone who would help them (*P* < 0.001). In their relations with medical institutions, they tended to understand the explanations given by their doctors about their disease (*P* = 0.054) and to receive up-to-date information during medical visits (*P* = 0.086). They also tended to enjoy a hobby (*P* = 0.070). On the other hand, people not having someone who will listen tended to join self-help patient groups (*P* = 0.056) and attending patient gatherings held by the public health center (*P* = 0.058). There was no difference in living with family or not and the severity of ADL. Thirty-five replied “spouse” about the person who will listen empathically to anxieties or troubles, and 17 replied “other member of their family” (including multiple answers). Nine replied “friends or other person.” Patients who had attended patient gatherings held by the public health center also tended to join self-help patient groups (*P* = 0.004). There were no differences in other items.

## 4. Discussion

The present study showed that the total WHOQOL of home care patients with intractable neurological disease was associated with “attending patient gatherings held by the public health center” and “having someone who will listen empathically to anxieties or troubles” as well as ADL. ADL was associated with physical health, while “having someone who will listen” was related with psychological well-being. Earlier studies have shown an association between QOL of patients with neurological diseases and the severity of ADL [4, 17]. Although the present study showed a similar relation between the total WHOQOL score and ADL, the relationship with ADL was found in the physical domain, but not in the psychological domain. The present findings may suggest that emotional support such as “having someone who will listen empathically to anxieties or troubles” is important for home care patients with intractable neurological diseases, regardless of the severity of ADL.

Previous studies have reported that QOL was linked with depression, fatigue, and anxiety among patients with neurological diseases [5, 18, 19]. A study has indicated that depression was a major contributor to the QOL scores of patients with Parkinson's disease [6]. Meanwhile, an association between QOL and the existence of confiding and emotional support has been reported among people with neuromuscular disorders [20], Parkinson's disease [14], and ALS patients [21]. It is, hence, considered that emotional supports from family, friends, and health professionals are important to improve their symptoms of depression and QOL [9, 22]. In the present study, people having someone who will listen were also more likely to have someone who helps whenever he or she is in trouble. These findings suggested that the presence of someone nearby who will provide emotional as well as physical support was a key factor in the QOL of patients with neurological diseases.

Most of the patients replied “spouse” or “other member of their family” about persons who will listen empathically to anxieties or troubles. A family member is one of the most important factors affecting the QOL of patients with neurological disease [23]. A previous study of ALS patients showed the importance of the presence of caring family as well as the availability of technical aids [24]. In the present study, most patients (91.9%) had lived with family, and 60.8% had been cared for by family members. However, about one-third (35.1%) of the present patients replied “no” to a question of “having someone who will listen empathically to anxieties or troubles.” These results may suggest that their family cannot necessarily be such an emotional supporter as a person who would listen empathically to anxieties of the patients, even if patients live with family. On the other hand, family caregivers may bear physical and psychological distress [25–27]. A study on family caregivers of patients with Parkinson's disease has reported an association between the caregiver's psychological burden and QOL of the patients [28]. We could not elucidate the psychological states and burden of their family, because we did not investigate their family. But family caregivers may have anxieties or troubles due to the burden of caring, and they would also need social supports.

The present results showed a close relationship between the total QOL score and “attending patient gatherings held by the public health center.” Such relations were encountered in the psychological domain, though the significance was borderline (*P* = 0.058). The public health center in the city under study holds gatherings regularly for patients with neurological diseases and their family to contact and communicate with each other. On these occasions, public health nurses counsel the patients and their families. Such gatherings may be good occasions to provide patients and their family with emotional support to improve their QOL. Reversely, it was also shown that some patients not having someone who will listen were more likely to participate in the gatherings. The patients and families may attend such gatherings to seek out someone who will listen empathically to them or where they can find companionship for physical and emotional help.

In the present study, “having a hospital which will admit you when your condition suddenly changes” was associated with QOL in the psychological domain and environment domain. Previous studies have reported that anxiety about a medical institute to be accepted in emergencies was associated with QOL [29] and that patients satisfied with their medical care tended to have higher QOL [2, 29]. A study of ALS patients reported that higher patient satisfaction was related to their feeling that the physician understood their feelings [30]. For home care patients with intractable neurological diseases, having a certain hospital for admission can provide a sense of ease, as a place that can deal with sudden changes or emergencies. Medical institutions and professionals are necessary for patients as a place of refuge and people who can give medical care and provide emotional supports.

There were some limitations in this study. The present study had only 74 (62%) subjects within a limited area. These results may not adequately reflect the general conditions of home care patients with neurological diseases in Japan. Second, neurological diseases of participants in this study were of several kinds, so the findings may have been potentially affected by the kind of neurological diseases. Moreover, their QOL was assessed using the WHOQOL-BREF, a general QOL assessment, though it is used to assess the QOL of people with some disease as well. Specific assessment items may be required in the case of individual neurological diseases. Finally, this was a cross-sectional study, but longitudinal assessments will also be warranted since neurological diseases gradually lead to deterioration.

## 5. Conclusion

The present study found that the QOL of home care patients with intractable neurological disease was associated with “attending patient gatherings held by the public health center” and “having someone who will listen empathically to anxieties or troubles” as well as ADL. The present findings suggested that having someone who will provide emotional support was important for home care patients with neurological diseases. Furthermore, patient gatherings held by the public health center were expected to provide patients with emotional support. The patients and their families may attend such gatherings to seek out someone who will listen empathically to them or where they can find companionship for physical and emotional help. Public health nurses may be able to use the gatherings to provide emotional support to patients.

## Figures and Tables

**Table 1:** Characteristics of patients with intractable neurological diseases (*N* = 74).

Age	63.9 ± 12.0
Sex	
Male	38 (51.4)
Female	36 (48.6)
Disease name	
Parkinson's disease	42 (56.8)
Amyotrophic lateral sclerosis	3 (4.1)
Spinocerebellar	10 (13.5)
Multiple sclerosis	7 (9.5)
Multiple system atrophy	4 (5.4)
Myasthenia gravis	3 (4.1)
Other	5 (6.8)
Age at disease onset	55.9 ± 13.6
Duration of illness (years)	7.95 ± 5.49
Living status	
Alone	6 (8.1)
With family	68 (91.9)
Caregiver	
Family members	45 (60.8)
Home care workers	7 (9.5)
Not having caregiver	22 (29.7)
ADL	
Independent group	37 (50.0)
Low-level care group	15 (20.3)
High-level care group	22 (29.7)
WHOQOL-BREF	
Total WHOQOL score	11.6 ± 2.4
Physical health (domain 1)	11.2 ± 3.2
Psychological well-being (domain 2)	11.5 ± 3.3
Social relationships (domain 3)	12.0 ± 3.2
Environment (domain 4)	12.0 ± 2.3

Data are expressed as frequency (%) and mean ± standard deviation (SD).

**Table 2:** Factors related to the total WHOQOL score (Low-score and high-score groups).

	Low-score group	High-score group		Adjusted sex, age, and ADL	
	(*N* = 39)	(*N* = 35)	OR	95% CI	*P* value
Living status					
Alone	2 (5.1)	4 (11.4)	0.59	0.08–4.21	0.600
With family	37 (94.9)	31 (88.6)			
ADL					
Independent group	17 (43.6)	5 (14.3)			
Low-level care group	8 (20.5)	7 (20.0)	2.19	1.19–4.01	0.011
High-level care group	14 (35.9)	23 (65.7)			
Hobby					
Not having	25 (64.1)	11 (32.4)	3.71	1.30–10.64	0.015
Having	14 (35.9)	23 (67.6)			
Frequency of going out					
Less than one time/week	12 (30.8)	3 (8.6)			
One time/week	7 (17.9)	5 (14.3)	1.98	0.97–4.05	0.060
Two times or more/week	20 (51.3)	27 (77.1)			
A person who will listen empathically to anxieties or troubles					
Not having	18 (47.4)	8 (23.5)	3.29	1.02–10.62	0.046
Having	20 (52.6)	26 (76.5)			
Someone who helps you whenever you are in trouble					
Not having	6 (15.4)	4 (11.4)	1.60	0.35–7.26	0.542
Having	33 (84.6)	31 (88.6)			
Self-help patient group					
Not join	33 (84.6)	26 (76.5)	1.76	0.49–6.24	0.385
Join	6 (15.4)	8 (23.5)			
Patient gatherings held by the public health center					
Not attending	31 (79.5)	20 (57.1)	3.83	1.19–12.34	0.025
Attending	8 (20.5)	15 (42.9)			
Up-to-date information during medical visits					
Not receiving	21 (53.8)	16 (45.7)	1.32	0.47–3.69	0.602
Receiving	18 (46.2)	19 (54.3)			
A hospital which will admit you when your condition suddenly changes					
Not having	14 (35.9)	6 (17.6)	2.31	0.71–7.56	0.165
Having	25 (64.1)	28 (82.4)			
An easy-to-understand explanation about their diseases					
Not receiving	7 (17.9)	7 (20.0)	0.61	0.17–2.20	0.447
Receiving	32 (82.1)	28 (80.0)			
Consultations with medical professionals during medical visits					
Not receiving	14 (35.9)	12 (35.3)	1.69	0.55–5.21	0.362
Receiving	25 (64.1)	22 (64.7)			
Home-visit nursing or rehabilitation service					
Not using	29 (74.4)	32 (91.4)	0.49	0.11–2.19	0.352
Using	10 (25.6)	3 (8.6)			
Home care services					
Not using	33 (84.6)	28 (80.0)	2.70	0.61–11.92	0.190
Using	6 (15.4)	7 (20.0)			

Data are expressed as frequency (%), odds ratio (OR), and 95% confidential interval (CI) of logistic regression analysis.

Odds ratio shows relation with the total QOL score (low-score and high-score groups) and each factor after adjusting for sex, age, and ADL, using multiple logistic regression analysis.

*P* value by multiple logistic regression analysis.

**Table 3:** Correlates of the total WHOQOL score (low- and high-score groups).

		Multiple adjustment	
	OR	95% CI	*P* value
ADL	2.17	1.06–4.43	0.033
Hobby	2.25	0.69–7.37	0.179
Frequency of going out	1.78	0.81–3.94	0.152
A person who will listen empathically to anxieties or troubles	6.38	1.31–30.99	0.022
Patient gatherings held by public health center	6.07	1.37–26.88	0.018

Data are expressed by involving odds ratio (OR) and 95% confidential interval (CI) of logistic regression analysis.

Odds ratio shows value turned on sex, age, ADL, a person who will listen empathically to anxieties or trouble, frequency of going out, not attending/attending patient gatherings held by the public health center, and not having/having hobby.

*P* value by multiple logistic regression analysis.

**Table 4:** Correlates of each domain of WHOQOL-BREF score (low- and high-score groups).

	Adjusted sex, age, and ADL		Multiple adjustment	
	OR^a^ (95% CI)	*P* value	OR^b^ (95% CI)	*P* value
Physical health (domain 1)				
ADL			2.57 (1.27–5.20)	0.009
A person who will listen empathically to anxieties or troubles			3.42 (0.98–11.92)	0.053
Psychological well-being (domain 2)				
ADL	—	—	1.60 (0.72–3.59)	0.249
Hobby	8.47 (2.44–29.47)	0.001	5.78 (1.31–25.52)	0.021
A person who will listen empathically to anxieties or troubles	3.42 (0.98–11.90)	0.054	8.79 (1.17–65.93)	0.034
A hospital which will admit you when your condition suddenly changes	3.34 (0.92–12.08)	0.066	7.66 (1.16–50.36)	0.034
Frequency of going out	2.10 (0.98–4.50)	0.057	1.87 (0.72–4.86)	0.196
Patient gatherings held by the public health center	3.18 (1.01–9.98)	0.048	5.00 (0.95–26.32)	0.058
Social relationships (domain 3)				
ADL	—	—	0.57 (0.24–1.39)	0.219
A person who will listen empathically to anxieties or troubles	4.76 (1.57–14.37)	0.006	4.17 (1.04–16.64)	0.043
Someone who helps you whenever you are in trouble	5.27 (1.17–23.81)	0.031	3.15 (0.53–18.72)	0.208
Home-visit nursing or rehabilitation service	0.04 (0.01–0.27)	0.001	0.04 (0.01–0.30)	0.001
Environment (domain 4)				
ADL	—	—	0.53 (0.26–1.08)	0.082
A hospital which will admit you when your condition suddenly changes	4.49 (1.36–14.76)	0.013	5.57 (1.29–24.00)	0.021
Up-to-date information during medical visits	2.31 (0.86–6.19)	0.097	2.62 (0.75–9.11)	0.131
Hobby	2.46 (0.93–6.53)	0.070	3.64 (1.10–12.08)	0.035
Self-help patient group	3.07 (0.84–11.20)	0.089	4.07 (0.88–18.93)	0.073

Data are expressed as odds ratio (OR) and 95% confidential interval (CI) of logistic regression analysis.

^a^Odds ratio shows relation with each domain of WHOQOL-BREF and each factor after adjusting for sex, age, and ADL.

^b^Odds ratio shows relation with each domain and the factors that tend to be related (*P *< 0.10) when adjusting for sex, age, and ADL.

*P* value by multiple logistic regression analysis.

**Table 5:** Relation with a person who will listen empathically to anxieties or troubles with the use of welfare resources and medical institutions.

	Have no person who will listen		Have person who will listen		*P* value
	*n*	%	*n*	%
Living status					
Alone	1	3.8	5	10.9	0.408
With family	25	96.2	41	89.1	
Hobby					
Not having	17	65.4	18	40.0	0.070
Having	9	34.6	27	60.0	
Patient gatherings held by the public health center					
Not attending	14	53.8	36	78.3	0.058
Attending	12	46.2	10	21.7	
Someone who helps you whenever you are in trouble					
Not having	9	34.6	1	2.2	<0.001
Having	17	65.4	45	97.8	
Self-help patient group					
Not join	18	69.2	40	88.9	0.056
Join	8	30.8	5	11.1	
Up-to-date information during medical visits					
Not receiving	17	65.4	19	41.3	0.086
Receiving	9	34.6	27	58.7	
An easy-to-understand explanation about their diseases					
Not receiving	8	30.8	5	10.9	0.054
Receiving	18	69.2	41	89.1	
Consultations with medical professionals during medical visits					
Not receiving	13	50.0	13	28.3	0.112
Receiving	13	50.0	33	71.7	

Data are expressed as frequency (%). *P* value by chi-square test.
